# Limitations of a metabolic network-based reverse ecology method for inferring host–pathogen interactions

**DOI:** 10.1186/s12859-017-1696-7

**Published:** 2017-05-25

**Authors:** Kazuhiro Takemoto, Kazuki Aie

**Affiliations:** Department of Bioscience and Bioinformatics, Kyushu Institute of Technology, Iizuka, Fukuoka 820-8502 Japan

**Keywords:** Reverse ecology, Metabolic networks, Species–species interactions, Systems biology

## Abstract

**Background:**

Host–pathogen interactions are important in a wide range of research fields. Given the importance of metabolic crosstalk between hosts and pathogens, a metabolic network-based reverse ecology method was proposed to infer these interactions. However, the validity of this method remains unclear because of the various explanations presented and the influence of potentially confounding factors that have thus far been neglected.

**Results:**

We re-evaluated the importance of the reverse ecology method for evaluating host–pathogen interactions while statistically controlling for confounding effects using oxygen requirement, genome, metabolic network, and phylogeny data. Our data analyses showed that host–pathogen interactions were more strongly influenced by genome size, primary network parameters (e.g., number of edges), oxygen requirement, and phylogeny than the reserve ecology-based measures.

**Conclusion:**

These results indicate the limitations of the reverse ecology method; however, they do not discount the importance of adopting reverse ecology approaches altogether. Rather, we highlight the need for developing more suitable methods for inferring host–pathogen interactions and conducting more careful examinations of the relationships between metabolic networks and host–pathogen interactions.

**Electronic supplementary material:**

The online version of this article (doi:10.1186/s12859-017-1696-7) contains supplementary material, which is available to authorized users.

## Background

Diseases spread in natural host (e.g., human and plant) populations via pathogens. Investigations of host–pathogen interactions are important not only in the context of basic scientific research but also in applied biological research fields such as medical science and disease ecology [1–3]. The development and progress of several new technologies and high-throughput methods have generated considerable host–pathogen interaction data, which have accumulated in several databases such as the Pathogen-Host Interactions database (PHI-base) [4] and Host Pathogen Interaction Database [5].

Elucidating the molecular mechanisms of host–pathogen interactions is important for host–pathogen interaction inference; in particular, pathogens use their biomolecules to hijack and re-wire numerous biochemical pathways in their hosts during infection [6]. Recognition of the importance of metabolic crosstalk between hosts and pathogens led to the proposal of a reverse ecology approach based on metabolic networks [7] as a computational framework for estimating host–pathogen interactions, which has attracted increasing attention [8]. Metabolism, a series of chemical reactions, is often represented as a network (known as a *metabolic network*). Metabolic networks have mainly been studied from a complex network perspective given the advances in network science [9, 10], especially network biology [11]. Indeed, many studies have evaluated adaptations to different environments (i.e., ecological interactions) by examining metabolic networks [12–14]. Specifically, Lévy et al. [15] used a graph theoretical algorithm to identify the set of exogenously acquired nutrients (known as a *seed set*) in metabolic networks, and proposed measures for estimating the cooperative interactions between a species pair [16, 17]: the biosynthetic support score (BSS) and the metabolic complementarity index (MCI). The BSS quantifies the metabolic ability of an organism (e.g., host) to meet the nutritional requirements of another organism (e.g., pathogen) [16]. The MCI indicates the degree of support one organism provides to another organism through biosynthetic complementarity (i.e., potential for syntrophism). Although the authors [16] stated that the MCI is particularly useful for estimating pairwise interactions between co-occurring microbes, it is also expected to be useful for assessing host–pathogen interactions because of the common occurrence of pathogenic symbiosis in plants [18] and insects [19]. A previous study [17] showed that these measures (particularly the BSS) were effective for predicting host–pathogen interactions. The reverse ecology method has been implemented as a software [16] and R-package [20], and has been applied in several microbial ecology studies such as studies of the human gut microbiome (e.g., [21, 22]).

However, more careful examination may be required to determine the importance of reverse ecology-based measures (i.e., BSS and MCI) on host–pathogen interaction inference. In particular, previous studies did not take several alternative factors into account. For example, genome size and total gene number were not directly evaluated, although it is well-known that these genomic parameters of pathogens are lower than those of free-living microbes [23]. The oxygen requirement of pathogens has also been omitted in previous models, despite the importance of oxygen in host–pathogen interactions [24] (i.e., pathogens exhibit remarkable adaptability and prevail in a wide range of oxygen concentrations); in addition, metabolic networks of aerobes are larger and less modular (or compartmentalized) than those of anaerobes [25, 26]. The effect of metabolic network modularity on host–pathogen interactions has not yet been evaluated, although previous studies [27, 28] showed that the metabolic network modularity of obligate host-associated bacteria was lower than that of free-living bacteria. In turn, genomic, physiological, and network parameters may influence the BSS and MCI values; thus, controlling for these potentially confounding effects is necessary to determine the importance and relevance of the BSS and MCI. However, previous studies did not control for these confounding effects. More importantly, the effects of phylogenetic signals were not considered, although the importance of phylogeny in evaluating associations between biological features has been well-established through comparative phylogenetic analyses [29, 30]. For example, an opposite conclusion may be derived when considering comparative phylogenetic analysis [31, 32].

Thus, we re-evaluated the contribution of the parameters BSS and MCI to pathogen/non-pathogen classification while statistically controlling for potentially confounding effects using data related to oxygen requirement, genome, and metabolic networks. We also performed comparative phylogenetic analyses to evaluate the effects of phylogenetic signals on the association between reverse ecology-based measures and host–pathogen interactions.

## Methods

### Host–pathogen interactions

Host–pathogen interaction data were downloaded from PHI-base (www.phi-base.org) [4] on July 28, 2016. Pathogenic species were chosen based on the availability of metabolic network data in the Kyoto Encyclopedia of Genes and Genomes (KEGG) database [33] and information related to oxygen requirement in the Microbial Physiology and Metabolism (MIPMET) database (takemoto08.bio.kyutech.ac.jp/mipmet/); 54 mammalian pathogens, 13 plant pathogens, and 15 insect pathogens were selected (Additional file 1). The classification of mammalian/plant/insect pathogens was defined based on the information of *Host Description* (i.e., host classification) for each pathogen in the XML file downloadable from PHI-base. Specifically, the host species of mammalian pathogens are categorized into *Rodents, Rabbits & Hares*, *Primates*, *Odd-toed Ungulates*, and *Even-toed Ungulates*. The host species of plant pathogens are classified into *Eudicots*, *Flowering Plants*, and *Monocots*. Host species insects are classified as *Bees*, *Beetles*, *Flies*, *Black-legged Ticks*, *Moths*, and *Fleas*.

### Non-pathogenic species

We defined 273 candidate non-pathogenic species based on microbial physiology and metabolism data (i.e., lifestyle, habitat, and growth temperature) (Additional file 2). Data related to microbial physiology and metabolism were collected from the literature (e.g., [25, 26, 34]) and are available in the MIPMET database. The datasets for microbial physiology and metabolism were downloaded from the database on August 25, 2016. We first selected species that were classified both as *Free-living* in the *Biotic* category and as *Mesophilic* in the *Temperature* category, while species classified as *Host-associated* in the *Habitat* category were ignored. We next removed species whose genera appeared in the PHI-base dataset. Finally, we only selected species whose oxygen requirement data were available in the database.

### Biosynthetic support score and metabolic complementarity index

The BSS and MCI values between species were calculated using NetCooperate software [16], downloaded from the website (depts.washington.edu/elbogs/NetCooperate/NetCooperateWeb.cgi) on September 2, 2016. The BSS is defined as the fraction of the seed set of an organism that is available in the metabolic network of another organism. The MCI is defined as the fraction of the seed set of an organism that is available in the non-seed set of another organism. Both the BSS and MCI range from 0 (no potential for cooperation) to 1 (perfect cooperation). The metabolic networks, required for the software, were constructed according to previous studies [17, 25]. XML files (version 0.7.1) containing metabolic network data (i.e., substrate–product relationships and reversibility/irreversibility of chemical reactions) were downloaded from the KEGG database [33] (ftp://ftp.genome.jp/pub/kegg/xml/kgml/metabolic/organisms/) on August 26, 2016. Based on the XML files, metabolic networks were represented as directed networks, in which the nodes and edges correspond to metabolites and reactions (i.e., substrate–product relationships), respectively. Because the use of such data may be desirable to ensure reproducibility, the present dataset on metabolic networks is available upon request. When calculating the BSS and MCI between hosts and microbes, we focused on representative host species whose metabolic pathways have been well-characterized using experimental approaches, because the metabolic networks of hosts registered in PHI-base may be not available in the KEGG database; specifically, we used the metabolic networks of *Homo sapiens* (human), *Arabidopsis thaliana* (thale cress), and *Drosophila melanogaster* (fruit fly) for mammal, plant, and insect host species, respectively. The BSS and MCI are asymmetric between a species pair [16] (i.e., host and microbe, in this study); thus, we considered two types of BSS and MCI values, respectively: we calculated scores for the biosynthetic support of a microbe for a host (BSS_MH_), biosynthetic support of a host for a microbe (BSS_HM_), biosynthetic complement of a microbe for a host (MCI_MH_), and biosynthetic complement of a host for a microbe (MCI_HM_).

### Genomic and network parameters

For microbes, we obtained the genome size and number of total protein-encoding genes from the KEGG database on October 30, 2016. As network parameters, we evaluated the number of nodes (*N*) and number of directed edges (*E*). We focused on network modularity, since a previous study [28] demonstrated its importance on pathogen/non-pathogen classification. The modularity of networks is often measured using the *Q*-value (e.g., [35]). *Q* is defined as the fraction of edges that lie within, rather than between, modules relative to that expected by chance. The *Q*-value is a size-invariant measure; thus, the role of network size on modularity can be analyzed as an independent topological variable of interest [28] (however, see [36]). A network with a higher *Q*-value indicates a higher modular structure. Thus, we need to find the global maximum *Q*-value over all possible divisions. Since it is hard to find the optimal division with the maximum *Q* in general, approximate optimization techniques are required. In this study, a spectral optimization method was used for directed networks [37, 38] to avoid the resolution limit problem in community (or module) detection [35, 39] as much as possible.

### Statistical analysis

To evaluate the contribution of each parameter (or factor) to pathogen/non-pathogen classification, we conducted logistic regression analyses using R software (version 3.3.2; www.R-project.org). There was no biological replicate in our dataset (see also Additional file 1). The ordinary logistic regression based on fixed effects was first considered, for which we constructed full models encompassing the given explanatory variables, and selected the best model based on the sample size-corrected version of Akaike information criterion (AICc) values using the R package *MuMIn* (version 1.15.6). The quantitative variables were normalized to the same scale, with a mean of 0 and standard deviation of 1, using the *scale* function in R before the analysis. We used the *power.roc.test* function in the R package *pROC* (version 1.9.1) to estimate the required sample size based on the area under the receiver operating characteristic curve (AUC) value of the best model, statistical power, and balance between control and case observations (i.e., non-pathogens and pathogens). To avoid model selection bias, we also adopted a model-averaging approach [40], from which we obtained the averaged models in the top 95% confidence set of models using the *model.avg* function in the R package *MuMIn*. Genome size and total gene number were log-transformed for all analyses.

To remove the effects of phylogenetic signals from the regression analyses, we performed phylogenetic logistic regression analyses using the function *phyloglm* in the R-package *phylolm* (version 2.5). The phylogenetic trees, which are required for phylogenetic regression, were constructed using 16S rRNA sequence data according to the all-species living tree project [41] (Additional files 3, 4 and 5). 16S rRNA gene sequences were obtained from the KEGG database on November 30, 2016. After multiple alignments of the nucleotide sequences using ClustalW2 software, the phylogenetic tree was constructed using NJplot (doua.prabi.fr/software/njplot). Similar to our approach for logistic regression analyses, we constructed full models and then selected the best model based on AICc values. We also obtained the averaged models.

The contribution (i.e., non-zero estimate) of each explanatory variable to the pathogen/non-pathogen dichotomy was considered to be complete when the associated *p*-value was less than 0.05.

## Results and Discussion

### Re-evaluation of the metabolic network-based reverse ecology method

The conditions for the present data analysis may differ from those used in the previous study [17]. For example, the pathogen and non-pathogen datasets may differ between this study and the previous study because the dataset was not clearly described in the previous study. Metabolic networks may also differ between this study and the previous study because the database has been updated. To determine whether the differences in analytical conditions were not limiting, we first evaluated the validity of the reverse ecology method under similar conditions as those used in the previous study; that is, we performed statistical analysis using only the BSS (BSS_HM_ and BSS_MH_) and MCI (MCI_HM_ and MCI_MH_) values. We then determined the contributions of the BSSs and MCIs to pathogen/non-pathogen classification (Table 1). Our results were similar to those of the previous study and were consistent with empirical evidence. In particular, biosynthetic support of hosts for microbes (BSS_HM_) was observed in host–pathogen interactions; however, biosynthetic support of microbes for hosts (BSS_MH_) was negatively or not associated with the interactions. This result reflects the parasitism of pathogens (i.e., pathogens benefit from hosts, while hosts do not benefit from pathogens). For plants and insects, the biosynthetic complement of microbes for hosts (MCI_MH_) was observed in the host–pathogen interactions because of pathogenic symbiosis in plants [18] and insects [19]. The biosynthetic complement of the hosts for microbes (MCI_HM_) showed a certain degree of negative contribution to the pathogen/non-pathogen classification. This indicates that pathogens avoid benefiting from hosts in the context of biosynthetic complementation. This result is puzzling; however, it may be explained as follows. MCI_HM_ is defined as the fraction of the seed set of a microbe that is available in the non-seed set of a host, whereas BSS_HM_ is the fraction of the seed set of the microbe available in all metabolites (i.e., union of the seed set and non-seed set) of the host. Thus, the negative effect of MCI_HM_ despite the positive effect of BSS_HM_ indicates that the seed set of the microbe is mainly supported by the seed set of the host. This suggests competition between hosts and microbes (i.e., microbes consume the nutrients required by the host), which is a parasitic property.

**Table 1:** Influences of reverse ecology-based measures on pathogen/non-pathogen classification

Variables	Mammalian pathogens		Plant pathogens		Insect pathogens	
	Estimate[Averaged]	Estimate[Best]	Estimate[Averaged]	Estimate[Best]	Estimate[Averaged]	Estimate[Best]
BSS_HM_	**1.505 (<0.01)**	**1.485 (<0.01)**	**2.058 (0.03)**	**2.347 (0.01)**	**2.356 (0.01)**	**2.592 (<0.01)**
BSS_MH_	0.086 (0.74)		0.51 (0.55)		**−1.787 (0.03)**	**−1.871 (0.02)**
MCI_HM_	**−1.191 (<0.01)**	**−1.201 (<0.01)**	−2.098 (0.06)	−2.124 (0.05)	**−2.135 (0.02)**	**−2.265 (0.01)**
MCI_MH_	0.083 (0.75)		**2.043 (<0.01)**	**2.134 (<0.01)**	**2.365 (0.01)**	**2.727 (<0.01)**
AICc		284.4		87.6		111.2

BSS_HM_ and BSS_MH_ correspond to the biosynthetic support score (BSS) of hosts for microbes and the BSS of microbes for hosts, respectively. MCI_HM_ and MCI_MH_ are the metabolic complement index (MCI) of hosts for microbes and the MCI of microbes for hosts, respectively. Estimates in the best and averaged models based on logistic regression are shown. Values in brackets indicate associated *p*-values. Values in bold indicate statistical significance. AICc denotes the sample size-corrected version of the Akaike information criterion value

### Effects of genomic, physiological, and network parameters

We aimed to confirm the contributions of the BSS and MCI to pathogen/non-pathogen classification. However, the validity of the BSS and MCI remains controversial; this is because of other factors that may dominantly contribute to pathogen/non-pathogen classification, as described in the Background section. Thus, we next constructed full models encompassing all explanatory variables (BSS_HM_, BSS_MH_, MCI_HM_, MCI_MH_, genome size, total gene number, oxygen requirement, *N*, *E*, and *Q*) to control for potentially confounding effects. The AICc values in the best models generally decreased because of the consideration of the physiological, genomic, and primary network parameters (Tables 1 and 2). This indicates the importance of consideration of these parameters. The averaged models showed that host–pathogen interactions were affected by the oxygen requirement (i.e., anaerobic or not) and primary network parameters (i.e., *N* and *E*) of microbial metabolic networks rather than by the BSS and MCI, although these metabolic network-based reverse ecology parameters were found to partly contribute to the best models (Table 2). This is partly because the BSS and MCI are strongly related to the other parameters. In mammalian pathogens, for example, BSS_HM_ is positively correlated with *N* (Spearman’s rank correlation coefficient *r*_*s*_ = 0.94, *p* < 2.2 × 10^−16^) and *E* (*r*_*s*_ = 0.94, *p* < 2.2 × 10^−16^). MCI_HM_ is also positively associated with with *N* (*r*_*s*_ = 0.84, *p* < 2.2 × 10^−16^) and *E* (*r*_*s*_ = 0.84, *p* < 2.2 × 10^−16^). Empirical evidence supports these results. In particular, mammalian pathogens are generally facultative or strictly aerobes. This is consistent with the observation that pathogens must adapt to varied oxygen concentrations [24]. Mammalian pathogens show smaller genome sizes, and both mammalian and insect pathogens have relatively smaller metabolic networks. This indicates the minimalism of pathogens [23]. However, the previous study [17] showed that the number of nodes had a limited effect on pathogen/non-pathogen prediction using receiver operating characteristic curves. This discrepancy between the present and previous study is related to the use of different analysis methods. The receiver operating characteristic-based analyses used in the previous study did not control for confounding effects; thus, the effect of the number of nodes was likely underestimated. Moreover, the previous study did not evaluate the effect of another primary network parameter: the number of edges. Pathogens have a relatively large number of directed edges, indicating that the metabolic networks of the pathogens are relatively dense. This may be because many metabolic pathways, except for central metabolism (such as energy metabolism), in pathogens depend on host species metabolism [42, 43]. Pathogens lack peripheral metabolic pathways (e.g., lipid metabolism and amino acid metabolism), which is supported by the importance of amino acids on host–pathogen metabolic interactions [8] and is consistent with the conclusion of a bioinformatics study on the pathway-based inference of host–pathogen interactions [44]. Metabolic networks exhibit a bow-tie (or core–peripheral) structure [45]: they can be decomposed into densely connected giant components (core) and thinly connected peripheral subnetworks. Central metabolism is located at the core; thus, metabolic networks of pathogens are denser than those of non-pathogens because they only consist of densely connected components. In contrast to the previous studies [27, 28], metabolic network modularity did not differ between pathogens (or host-associated species) and non-pathogens, which is in line with the conclusion of other studies. In particular, the size of the metabolic network is a major determinant of network modularity [46]; that is, the difference in metabolic networks between pathogens and non-pathogens is explained by network size (i.e., *N* and *E*) rather than network modularity. Furthermore, the previously observed difference in network modularity between host-associated species and free-living species was probably due to a lack of available data on metabolic reactions; rather, metabolic network modularity was found to be dependent on species growth conditions such as oxygen requirement [47]. These previous studies also support the importance of the oxygen requirement and primary network parameters. However, it remains possible that the observed limited effect of the BSS and MCI is due to the sample size; in particular, our dataset contained only 13 plant pathogens and 15 insect pathogens; thus, statistical power for detecting an effect may be low. However, the AUC values obtained from the best models in the cases of plant pathogens and insect pathogens were relatively high at 0.844 and 0.905, respectively. When the statistical power of 0.95 was considered, the required sample sizes of plant pathogens and insect pathogens were 9 and 6, respectively. This result indicates that the sample sizes pose few problems.

**Table 2:** Influence of explanatory variables on pathogen/non-pathogen classification when considering genomic, physiological, and network parameters in addition to reverse ecology-based measures

Variables	Mammalian pathogens		Plant pathogens		Insect pathogens	
	Estimate[Averaged]	Estimate[Best]	Estimate[Averaged]	Estimate[Best]	Estimate[Averaged]	Estimate[Best]
BSS_HM_	0.841 (0.29)		2.17 (0.08)	**2.347 (0.01)**	0.133 (0.90)	
BSS_MH_	−0.838 (0.35)		0.674 (0.60)		−1.042 (0.41)	1.129 (0.06)
MCI_HM_	−0.143 (0.86)		−1.758 (0.19)	−2.124 (0.05)	0.534 (0.53)	
MCI_MH_	−0.696 (0.07)	**−0.752 (0.03)**	**1.819 (0.01)**	**2.134 (<0.01)**	1.207 (0.10)	
Genome size	−1.951 (0.16)	**−2.621 (0.04)**	0.657 (0.73)		−1.326 (0.47)	
#Genes	1.631 (0.29)	1.812 (0.15)	−1.193 (0.54)		0.561 (0.80)	
Oxygen	**−1.726 (<0.01)**	**−1.646 (0.01)**	−16.211 (0.99)		−18.171 (0.99)	−18.264 (0.99)
*N*	**−4.564 (<0.01)**	**−4.874 (<0.01)**	−0.615 (0.78)		**−7.541 (<0.01)**	**−8.816 (<0.01)**
*E*	**5.552 (<0.01)**	**5.79 (<0.01)**	1.64 (0.33)		**7.181 (<0.01)**	**7.213 (<0.01)**
*Q*	−0.307 (0.17)		0.367 (0.33)		−0.656 (0.09)	−0.577 (0.10)
AICc		241.7		87.6		90.3

The variable “Oxygen” indicates the species oxygen requirement (i.e., anaerobe or not). *N* and *E* correspond to the number of nodes and number of directed edges, respectively. *Q* indicates network modularity. See the footnote to Table 1 for a description of all other table elements

### Effect of phylogenetic signals

As described in the Background, it is important to consider the effects of phylogenetic signals when investigating the associations between biological features. We removed the phylogenetic effects using phylogenetic logistic regression. The AICc values in the best models generally decreased with consideration of the phylogeny (Tables 2 and 3), indicating the importance of phylogeny. Again, the averaged models revealed the limited effects of the BSS and MCI on pathogen/non-pathogen classification (Table 3). Moreover, the averaged models showed that the other parameters were only minimally associated with host–pathogen interactions; however, clear contributions of primary network parameters (i.e., *N* and *E*) were observed in the case of insect pathogens. According to the best models, each parameter partly contributes to pathogen/non-pathogen classification. For example, the genome sizes of mammalian pathogens were smaller than those of non-pathogens, and the metabolic networks of mammalian pathogens were denser than those of non-pathogens. In addition, biosynthetic complementation of the microbes for the insect host was observed. Insect pathogens are typically aerobic. However, the averaged models showed that these results were not statistically robust. The difference between the best model and averaged model was due to model selection bias. These results indicate phylogenetic bias in host–pathogen interactions (i.e., phylogenetic information, rather than reverse ecology-based measures and other parameters, determines whether a species is pathogenic). The effect of phylogenetic signals (the fact that important biological associations were not conclusively determined with phylogenetic correction) has been observed in a wide range of research fields (e.g., in metabolic networks [31] and in species–species interactions in food webs [32]). However, more careful examinations are required because of the limitations of phylogenetic comparative analysis. In particular, phylogenetic comparative analysis assumes a Brownian motion-like evolution of biological traits on a phylogenetic tree with accurate branch lengths, and thus may result in misleading conclusions. We constructed the phylogenetic trees based on 16S rRNA sequences only to reduce computational costs. Ideally, a highly resolved phylogenic tree [48] constructed based on a common protein set across organisms may be required. In addition, statistical power decreases when a dataset is reduced in size following phylogenetic corrections [49]. As mentioned in the previous section, our dataset contained only a few samples for plant pathogens and insect pathogens; thus, statistical power may have been low. Ideally, the sample sizes required for suitable statistical power would be evaluated. However, methods for estimating the sample sizes have not yet been established for the phylogenetic logistic regression model. Thus, more careful examinations are required to determine the limited effect of the BSS and MCI. In this context, a larger dataset of host–pathogen interactions should be evaluated. The development of high-throughput sequencing techniques will enable the collection of such data. For example, metagenomic techniques can now reveal host–pathogen interactions [50]. Similar to numerous previous studies of host–pathogen interactions, our study was limited because of the lack of availability of accurate datasets for non-pathogenic species (i.e., negative set) owing to the lack of experimental evidence, although we avoided this limitation as much as possible by using data related to microbial physiology and metabolism. Metagenomic techniques may also enable acquisition of a more accurate dataset.

**Table 3:** Influences of explanatory variables on pathogen/non-pathogen classification when removing the effects of phylogenetic signals

Variables	Mammalian pathogens		Plant pathogens		Insect pathogens	
	Estimate[Averaged]	Estimate[Best]	Estimate[Averaged]	Estimate[Best]	Estimate[Averaged]	Estimate[Best]
BSS_HM_	0.558 (0.31)	0.448 (0.15)	−0.08 (0.92)		0.531 (0.67)	
BSS_MH_	−0.531 (0.40)		−0.164 (0.86)		−1.309 (0.37)	−1.494 (0.12)
MCI_HM_	−0.126 (0.83)		−0.826 (0.37)		0.346 (0.76)	
MCI_MH_	−0.269 (0.44)		0.592 (0.31)		1.159 (0.17)	**1.53 (0.03)**
Genome size	−0.805 (0.25)	**−0.644 (0.04)**	0.767 (0.61)		−0.973 (0.45)	
#Genes	0.005 (1.00)		−0.642 (0.71)		−0.049 (0.98)	
Oxygen	−0.486 (0.23)	−0.483 (0.20)	−5.029 (0.99)		−3.849 (0.99)	**−2.864 (0.04)**
*N*	0.309 (0.81)		−0.078 (0.96)	**1.085 (0.02)**	**−5.549 (0.03)**	**−4.766 (0.01)**
*E*	1.023 (0.17)	**0.865 (0.04)**	1.398 (0.32)		**5.288 (0.03)**	**4.711 (<0.01)**
*Q*	−0.047 (0.73)		0.274 (0.33)		−0.547 (0.14)	−0.539 (0.12)
AICc		199.6		68.4		91.2

See the footnotes to Tables 1 and 2 for descriptions of table elements. Estimates in the best and averaged models based on phylogenetic logistic regression are shown

## Conclusions

The results presented herein call into question the importance of the current version of the metabolic network-based reverse ecology approach (i.e., BSS and MCI) for host–pathogen interaction inference. Metabolic networks are still not fully understood in detail. For example, enzyme promiscuity [51], which implies that enzymes can catalyze multiple reactions, act on more than one substrate, or exert a range of suppressions (in which enzymatic function is suppressed by over-expressing enzymes with originally different functions [52]), suggests the existence of many hidden metabolic reactions. Consideration of these hidden metabolic reactions is important for understanding metabolic interactions in ecosystems. However, the results of the present study do not entirely discount the metabolic network-based reverse ecology approach; rather, these findings emphasize the need for developing more suitable methods for estimating host–pathogen interactions. For example, the definition of seed sets is controversial. Previous studies [15, 17] used a strongly connected component decomposition algorithm to identify a seed set. However, this method only focuses on network topology and does not consider biochemically feasible reactions. For example, it may be necessary to identify seed sets based on an algorithm of network expansion to generate the set of all possible metabolites that can be produced from a set of compounds, similar to the approach adopted in a previous study [53]. An approach for pathway-based inference of host–pathogen interactions [44] would also be useful, which would allow for more careful comparisons of metabolic networks between hosts and pathogens. Moreover, there are several metabolic models based on flux balance analysis. Originally, flux balance analysis was used to model metabolic processes in single species; however, in recent years, this method has started to be applied in microbial ecology (e.g., to examine the cooperative and competitive dynamics between different species) [54–58]. These methods can improve understanding of interspecies interactions at the metabolic level, although the computational costs are higher compared to those required with the reverse ecology method. Metabolic network-based reverse ecology remains a challenging research topic in the post-genomic era because of the importance of the human microbiome [59] and the earth microbiome [60]; thus, more careful investigations of the relationships between metabolic networks and host–pathogen interactions are needed.

## Additional files

Additional file 1: List of pathogens used in this study. This table shows the species name, Kyoto Encyclopedia of Genes and Genomes (KEGG) organism identifier (see www.genome.jp/kegg/catalog/org_list.html), host classification, oxygen requirement, genome size [bp], number of total genes [count], BSS_HM_, BSS_MH_, MCI_HM_, MCI_MH_, number of nodes, number of directed edges, and network modularity *Q* (XLSX 45 kb).

Additional file 2: List of non-pathogens used in this study. See the Additional file 1 caption for a description of table elements (XLSX 65 kb).

Additional file 3: Phylogenetic tree of mammalian pathogens and non-pathogens used in this study. Node labels correspond to the KEGG organism identifier. The tree is presented in the Newick format (TXT 6 kb).

Additional file 4: Phylogenetic tree of plant pathogens and non-pathogens used in this study. See the Additional file 3 caption for a detailed description (TXT 6 kb).

Additional file 5: Phylogenetic tree of insect pathogens and non-pathogens used in this study. See the Additional file 3 caption for a detailed description (TXT 6 kb).

## Abbreviations

AICc: Sample size-corrected version of Akaike Information Criterion
AUC: Area Under the receiver operating characteristic Curve
BSS: Biosynthetic support score
BSS_HM_: Biosynthetic support score of a host for a microbe
BSS_MH_: Biosynthetic support score of a microbe for a host
E: number of edges
KEGG: Kyoto encyclopedia of genes and genomes
MCI: Metabolic complementarity index
MCI_HM_: Biosynthetic complement index of a host for a microbe
MCI_MH_: Biosynthetic complement index of a microbe for a host
MIPMET: MIcrobial Physiology and METabolism
N: Number of nodes
PHI-base: Pathogen-host interactions database
